# Mortality and Causes of Death After Liver Transplantation: Analysis of Sex Differences in a Large Nationwide Cohort

**DOI:** 10.3389/ti.2022.10263

**Published:** 2022-05-09

**Authors:** M. Trinidad Serrano, Sergio Sabroso, Luis M. Esteban, Marina Berenguer, Constantino Fondevila, Sara Lorente, Luis Cortés, Gloria Sanchez-Antolin, Javier Nuño, Gloria De la Rosa, Magdalena Salcedo

**Affiliations:** ^1^ Digestive Diseases Department, Lozano Blesa University Hospital, Zaragoza, Spain; ^2^ Aragón Health Research Institute (IIS Aragón), Zaragoza, Spain; ^3^ Genetic and Molecular Epidemiology Group (GMEG), Spanish National Cancer Research Center (CNIO), Madrid, Spain; ^4^ Department of Applied Mathematics, Escuela Universitaria Politécnica La Almunia, University of Zaragoza, Zaragoza, Spain; ^5^ Hepatology and Liver Transplant Unit, La Fe University Hospital, La Fe Health Research Institute (IIS La Fe), Universidad de Valencia, Valencia, Spain; ^6^ Centro de Investigación Biomédica en Red de Enfermedades Hepáticas y Digestivas Ciberehd, Madrid, Spain; ^7^ Department of General and Digestive Surgery, Institut de Malalties Digestives I Metabòliques (IMDiM), Hospital Clínic, University of Barcelona, Barcelona, Spain; ^8^ Digestive Diseases Department, Rio Hortega University Hospital, Valladolid, Spain; ^9^ Department of General and Digestive Surgery, Ramon y Cajal University Hospital, Madrid, Spain; ^10^ Organización Nacional de Trasplantes (ONT), Secretaria Del Registro Español de Trasplante Hepático (RETH), Madrid, Spain; ^11^ Digestive Diseases Department, Gregorio Marañón University Hospital, Complutense University of Madrid, Zaragoza, Spain

**Keywords:** liver transplantation, mortality, survival, sex differences, cause of death

## Abstract

In the last few years, several studies have analyzed sex and gender differences in liver transplantation (LT), but none have performed a disaggregated analysis of both mortality and causes of death. Data from 15,998 patients, 11,914 (74.5%) males and 4,069 (25.5%) females, transplanted between 2000 and 2016 were obtained from the Liver Transplantation Spanish Registry. Survival analysis was applied to explore recipient sex as a risk factor for death. The causes of death at different follow-up duration were disaggregated by recipient sex for analysis. Short-term survival was higher in males, whereas long-term survival was higher in females. Survival at 1, 5 and 10 years post-transplant was 87.43%, 73.83%, and 61.23%, respectively, in males and 86.28%, 74.19%, and 65.10%, respectively, in females (*p* = 0.05). Post-LT mortality related to previous liver disease also presented sex differences. Males had 37% increased overall mortality from acute liver failure (*p* = 0.035) and 37% from HCV-negative cirrhosis (*p* < 0.001). Females had approximately 16% increased mortality when the liver disease was HCV-positive cirrhosis (*p* = 0.003). Regarding causes of death, non-malignancy HCV+ recurrence (6.3% vs. 3.9% of patients; *p* < 0.001), was more frequently reported in females. By contrast, death because of malignancy recurrence (3.9% vs. 2.2% of patients; *p* = 0.003) and *de novo* malignancy (4.8% vs. 2.5% of patients; *p* < 0.001) were significantly more frequent in male recipients. Cardiovascular disease, renal failure, and surgical complications were similar in both. In summary, male patients have lower short-term mortality than females but higher long-term and overall mortality. In addition, the post-LT mortality risk related to previous liver disease and the causes of mortality differ between males and females.

## Graphical Abstract

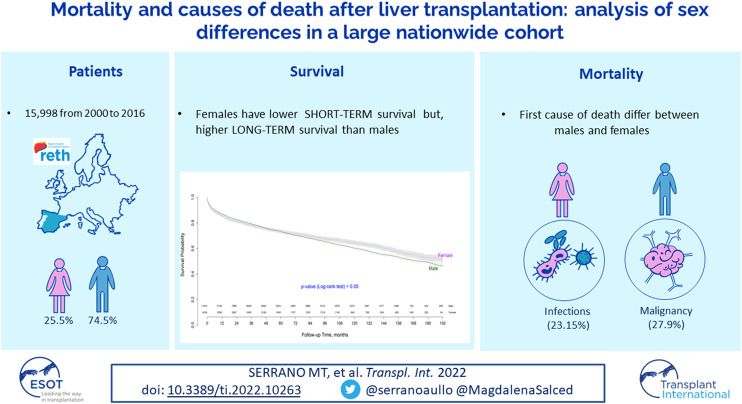

## Introduction

Liver transplantation (LT) is the best treatment for patients with end-stage liver disease. Advances in surgical techniques and medical management of patients have markedly improved outcomes. However, short-term mortality remains at 10%–15%, and no clear improvement in long-term mortality has been achieved in the last few years (1). Interest in causes of mortality after LT and how they vary with time is increasing. Boganate et al. (2) described short-term mortality occurring mainly due to infections and circulatory disease in the first 90 days after LT. Regarding long-term mortality, Watt et al. (3) analyzed a large cohort of patients and concluded that the most frequent causes of death were graft failure, malignancy, cardiovascular disease, and kidney failure. Subsequent studies corroborated these findings and, in recent years, special programs have been launched for the early detection and prevention of cardiovascular and cancerous diseases (4,5).

Sex- and gender-disaggregated data analyses are important for reducing health inequities in medicine and many recent studies have analyzed sex and gender differences in liver disease. For example, sex imbalances in MELD predictor, waiting list mortality, and survival after LT have been studied (6–8). However, no studies have analyzed mortality after LT from the perspective of sex and gender. Gathering sex-disaggregated mortality data could provide even greater insight into differential outcomes that are associated with biological differences and behaviors linked to gender norms. These studies are very likely essential to improving outcomes and developing sex and gender-specific short- and long-term risk prevention policies.

Sex depends on biological attributes and gender refers to social roles, behaviors and constructed identities. Given that databases typically collect only sex-related data, we will focus our analysis on sex differences. Therefore, the objective of this study was to analyze sex differences in short- and long-term mortality after LT in a large cohort of patients with long-term follow-up. In addition, we analyzed the specific causes of death from the perspective of sex recipient and calculated the cumulative incidence of mortality from specific causes in relation to the follow-up.

## Patients and Methods

### Study Design and Population

We retrospectively explored data collected from the Spanish Liver Transplant Registry (Registro Español de Trasplante Hepático, RETH). RETH is a multicenter registry that recruits data from all liver transplant units in Spain with periodic auditing. The inclusion criteria were transplants performed on patients older than 16 years, from January 2000 to December 2016 with follow-up to November 2017. Multi-visceral transplantations were excluded.

Data from the 15,998 liver transplant recipients were stratified by sex on the characteristics age of recipient, MELD, donor sex, age of donor, number of transplants, type of transplant, cold ischemia time, presence of hepatitis C virus (HCV), presence of HIV, and main liver disease (acute liver failure, cholestasis, cirrhosis HCV+, cirrhosis HCV-, liver cancer, or other causes).

Causes of death were captured in the post-transplant period, and the number of deaths in different periods were stratified by sex and cause. Causes of death were classified into the following categories: surgical complications, infections, recurrence of HCV-positive liver disease, recurrence of HCV-negative liver disease, tumor recurrence, *de novo* malignancy, circulatory disease, kidney failure, *de novo* liver disease, rejection, and others.

### Statistical Analysis

Data were descriptively analyzed; continuous variables were summarized as median and interquartile range and categorical variables as absolute and relative frequencies. Significant differences by sex of the recipient were established by the Mann-Whitney or chi-squared test as appropriate.

Survival analysis was applied to analyze recipient sex as a risk factor for overall mortality. Kaplan-Meier curves and log-rank tests were used to study the differences between male and females recipients. Regarding predictors of mortality, we used univariate and multivariate Cox proportional regression models to estimate the hazard ratios and 95% confidence intervals for prognostic variables in order to predict 1 month (early), 1 year (short-term), 5 years (long-term) and overall mortality. We also performed a sub-analysis to study differences between the sexes by main disease (acute liver failure, cholestasis, cirrhosis, cirrhosis HCV-positive, cirrhosis HCV-negative, liver cancer, or other causes), sex and age of the recipient, MELD, sex and age of the donor, and the presence of HIV in the recipient in order to predict mortality. The significance of the differences between male and female recipients was determined by a test of proportions.

The causes of death at different follow-up duration (from 1 to 10 years) were analyzed overall and disaggregated by recipient sex.

In addition, the relationship between cause of mortality and main disease was analyzed using a heatmap showing the correlation between groups of both variables by sex.

Analyses were performed using R v.4.0.3 (The R Foundation for Statistical Computing, Vienna, Austria).

## Results

Clinical characteristics of the recipients by sex are shown in Table 1. Our dataset consisted of 11,914 (74.5%) males and 4,069 (25.5%) females, with a longer median follow-up in females (4.6 years vs 4.2, *p* = 0.009). The median age of patients at time of LT [55 (IQR 49–61) and 56 (IQR 46–62) years, respectively] was not different. Donor sex was predominantly male (61.1%) among male recipients and female (50.2%) among female recipients (*p* < 0.001). Donation after circulatory death was infrequent in this series (1.1% of male and 0.9% of female; *p* = 0.282). The median donor age was significantly lower for female recipients than for male recipients (55 (IQR 40–68) vs. 57 (IQR 43–69) years, *p* < 0.001) and the MELD value at LT was slightly higher for females than males) (18 (IQR 12–22) vs. 17 (IQR 12–21), *p* = 0.022). A total of 860 (7.8%) men and 359 (8.8%) women received more than one liver transplant (*p* = 0.003). Regarding the urgency of the procedure, 5.8% and 12.1% of liver transplant procedures were urgent in males and female, respectively (*p* < 0.001). The median cold ischemia time was 365 min (IQR 290–471) in males and 360 min (IQR 280–470) in females (*p* = 0.007). More males than females had HIV infection (2.47% vs 1.65%; *p* = 0.003), but no differences in HCV-related liver disease were found. Differences were found in the distribution of the main liver disease (Table 1). The most frequent diseases were HCV-positive liver cirrhosis in women and HCV-negative liver cirrhosis in men.

**TABLE 1:** Clinical characteristics of the patients by sex.

Feature/sex	Male (*N* = 11,914)	Female (*N* = 4,069)	*p*-value
Follow-up, years			0.009
Median (IQR)	4.189 (1.170–8.832)	4.630 (1.109–9.663)	
Age of recipients, years			0.349
Median (IQR)	55 (49–61)	56 (46–62)	
MELD			0.022
Median (IQR)	17 (12–21)	18 (12–22)	
Donor sex			<0.001
Male	4,624 (38.88%)	2,022 (49.79%)	
Female	7,270 (61.12%)	2,039 (50.21%)	
Age of donors			<0.001
Median (IQR)	57 (43–69)	55 (40–68)	
Number of transplants			0.004
1	11,054 (92.78%)	3,710 (91.18%)	
2	801 (6.72%)	326 (8.01%)	
3	54 (0.45%)	31 (0.76%)	
4	5 (0.04%)	2 (0.05%)	
Type of transplant			
Elective	11,102 (94.18%)	3,551 (87.94%)	<0.001
Urgent	686 (5.82%)	487 (12.06%)	
Ischemia time, minutes			0.007
Median (IQR)	365 (290–471)	360 (280–470)	
HCV	4,349 (39.06%)	1,460 (38.37%)	0.460
HIV	294 (2.47%)	67 (1.65%)	0.003
Main disease			<0.001
Acute liver failure	273 (2.49%)	385 (10.36%)	
Cholestasis	293 (2.67%)	514 (13.83%)	
HCV-positive Cirrhosis	2,585 (23.60%)	991 (26.67%)	
HCV-negative Cirrhosis	4,149 (37.87%)	815 (21.93%)	
Liver cancer	3,314 (30.25%)	711 (19.13%)	
Other	341 (3.11%)	300 (8.07%)	

### Survival Analysis

**FIGURE 1:**
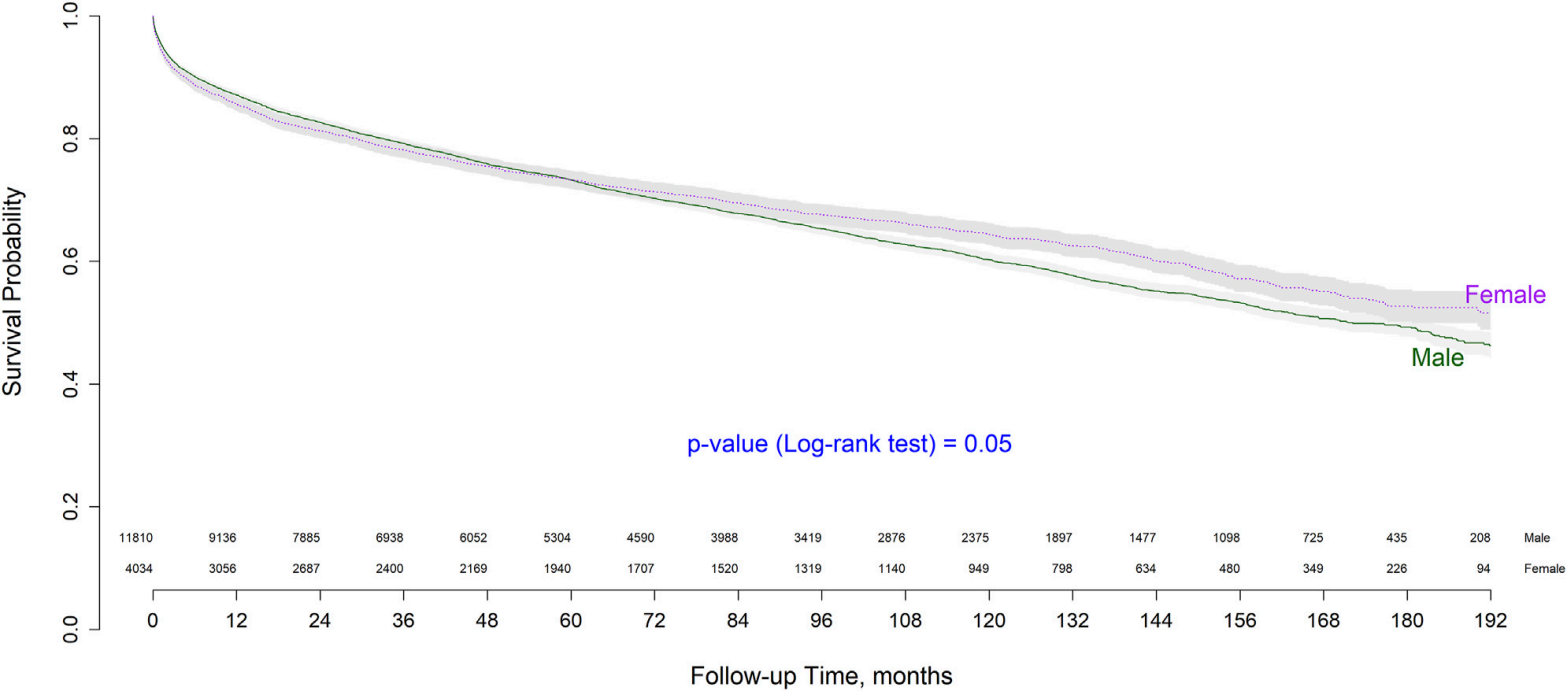
Kaplan-Meier overall survival curve by sex.

Patient survival according to recipient sex showed small differences in the short- and long-term. Short-term survival was higher in males, whereas overall and long-term survival were higher in females. Male survival at 1, 5, and 10 years post-transplant was 87.43%, 73.82%, and 61.23%, respectively, while that of female patients was 86.28%, 74.20%, and 65.10%, respectively. Sex-based survival probability after transplant is depicted as a Kaplan-Meier curve in Figure 1, which also provides the number of patients at risk. As shown, survival curves intersect in the follow-up period and the log-rank test shown no statistical significant differences between groups (*p* = 0.05).

**FIGURE 2:**
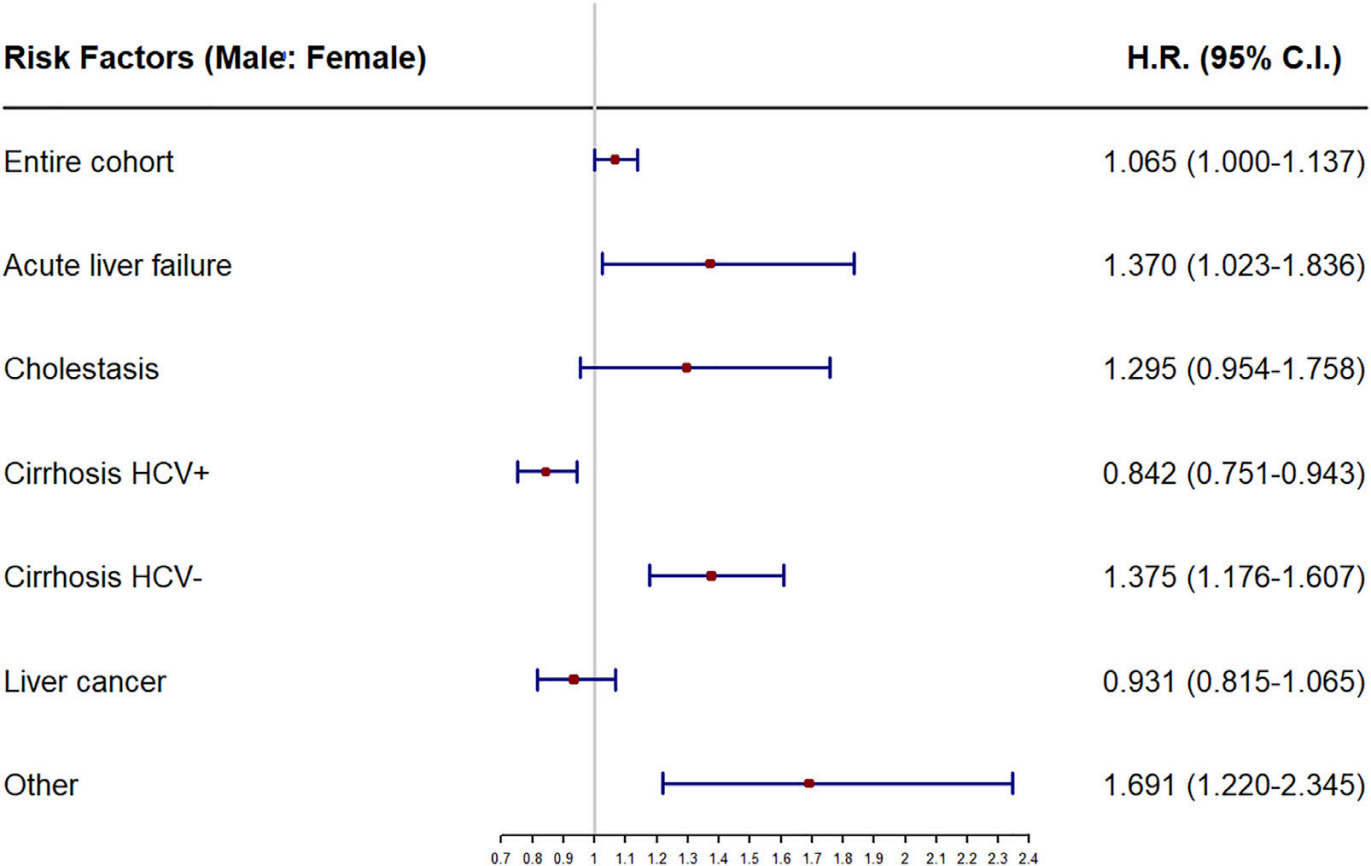
Forest plot of recipient sex as prognostic factor of overall mortality for the entire cohort and the subcohorts stratifying by main disease. HR, hazard ratios.

The analyses of recipient sex as a risk factor for mortality, or stratified by main disease are shown in Table 2. Female sex was found to be a risk factor for early (HR = 1.219, *p* = 0.019) and short term mortality (HR = 1.131, *p* = 0.014), while male was a risk factor (HR = 1.065, *p* = 0.050) for overall mortality, specifically when the main disease was acute liver failure (HR = 1.370, *p* = 0.035) and HCV-negative cirrhosis (HR = 1.375, *p* < 0.001). Male sex was a protective factor (HR = 0.884, *p* = 0.014), particularly when the main liver disease was HCV-positive cirrhosis (HR = 0.759; *p* = 0.002), In all other main liver diseases, no significant values were obtained. All of these results are depicted in detail in Table 2 and the forest plot in Figure 2.

**TABLE 2:** Survival analysis according to recipient sex considering the main liver diseases.

Risk factor	1-month hazard ratio (95% CI)	*p*-value	1-year hazard ratio (95% CI)	*p*-value	5-year hazard ratio (95% CI)	*p*-value	Overall hazard ratio (95% CI)	*p*-value
Sex (male:female)	0.820 (0.695–0.968)	0.019	0.884 (0.802–0.975)	0.014	0.972 (0.902–1.046)	0.445	1.065 (1.000–1.137)	0.050
Main disease (male:female)								
Acute liver failure	1.811 (1.149–2.857)	0.011	1.511 (1.061–2.152)	0.022	1.549 (1,123–2,134)	0.008	1.370 (1.023–1.836)	0.035
Cholestasis	1.419 (0.821–2.452)	0.210	1.345 (0.879–2.058)	0.171	1.299 (0.911–1,852)	0.148	1.295 (0.954–1.758)	0.096
HCV positive-Cirrhosis	0.914 (0.657–1.272)	0.594	0.759 (0.638–0.902)	0.002	0.788 (0.692–0.897)	0.003	0.842 (0.751–0.943)	0.003
HCV negative-Cirrhosis	1.132 (0.740–1.730)	0.568	1.153 (0.902–1.472)	0.256	1.223 (1,011–1,478)	0.038	1.375 (1.176–1.607)	<0.001
Liver cancer	0.665 (0.427–1.034)	0.070	0.803 (0.637–1.010)	0.061	0.867 (0.744–1,010)	0.068	0.931 (0.815–1.065)	0.299
Other	1.623 (0.803–3.280)	0.177	1.782 (1.13–2.805)	0.013	1.796 (1,233–2,615)	0.002	1.691 (1.220–2.345)	0.016

Comparison of 1-month, 1-year, 5-year, and overall.

Regarding the interaction between sex of recipient and MELD, urgent transplantation, donor age, recipient age, cold ischemia time, and HIV positivity, results are shown in Table 3. MELD score was a predictive risk factor for early and overall mortality (HR = 1.030, *p* = 0.014 and HR = 1.013, *p* = 0.017, respectively), but the interaction of MELD with recipient sex was not significant, thus we did not found differences by sex in the association of MELD with mortality.

**TABLE 3:** Interaction of main risk factors with recipient sex.

Risk factor	1-month HR (95%CI)	*p*-value	1-year HR (95%CI)	*p*-value	5-year HR (95%CI)	*p*-value	Overall HR (95%CI)	*p*-value
Age of recipient	0.986 (0.994–1.003)	0.121	1.010 (1.006–1.015)	<0.001	1.016 (1.012–1.020)	<0.001	1,021 (1.018–1.025)	<0.001
Age of recipient: Sex (female: male)	1.004 (1.001–1.007)	0.011	1.002 (1.001–1.004)	0.007	1.001 (1.000–1.002)	0.211	0,999 (0.997–1.000)	0.106
MELD	1.030 (1.006–1.055)	0.014	1.026 (1.012–1.040)	<0.001	1.015 (1.005–1.027)	0.006	1.013 (1.001–1.024)	0.017
MELD: Sex (female: male)	1.012 (0.995–1.030)	0.164	1.003 (0.992–1.013)	0.621	0.997 (0.988–1.006)	0.473	0.998 (0.989–1.006)	0.563
Age of donor	0.998 (0.994–1.003)	0.508	1.005 (1.003–1.008)	<0.001	1.008 (1.006–1.010)	<0.001	1.010 (1.008–1.012)	<0.001
Age of donor: Sex (female: male)	1.004 (1.001–1.007)	0.006	1.002 (1.001–1.004)	0.004	1.001 (1.000–1.003)	0.045	1 (0.998–1.001)	0.633
HIV (positive: negative)	0.923 (0.508–1.678)	0.793	0.932 (0.641–1.360)	0.714	1.080 (0.882–1.323)	0.455	1.266 (1.038–1.544)	0.020
HIV = positive: Sex (female: male)	1.174 (0.328–4.209)	0.805	1.056 (0.461–2.418)	0.897	0.735 (0.445–1.217)	0.232	0.769 (0.476–1.243)	0.284
Type of transplant (urgent: elective)	0.971 (0.612–1.542)	0.900	1.123 (0.879–1.434)	0.353	1.167 (1.012–1.340)	0.029	1.571 (1.386–1.780)	<0.001
Type of transplant = urgent: Sex (female: male)	2.173 (1.219–3.876)	0.009	1.311 (0.932–1.844)	0.120	1.072 (0.875–1.313)	0.502	0.662 (0.538–0.816)	<0.001
Ischemia time	1.001 (1–1.001)	<0.001	1 (1–1.001)	0.002	1 (1–1.001)	<0.001	1.0003 (1–1,001)	<0.001
Ischemia time: Sex (female: male)	1 (1–1.001)	0.081	1 (1–1.001)	0.057	1 (0.999–1)	0.542	0.999 (0.998–1)	0.067

Each row shows the hazard ratio (HR) associated with each risk factor, and below is the HR added to that risk factor because of an interaction with recipient sex.

Other potential risk factors and their interaction with recipient sex were also analyzed. Recipient sex showed a significant interaction with the age of the recipient (HR = 1.004, *p* = 0.011), age of the donor (HR = 1.004, *p* = 0.006) and urgency of transplant (HR = 2.173, *p* = 0.009) on early mortality. Regarding overall mortality we only found a significant interaction with recipient sex in the urgency of transplant (HR = 0.662, *p* < 0.001). All of these results are depicted in detail in Table 3.

In the multivariate analysis for the predictors of overall mortality (Table 4), recipient and donor age, number of transplants and the presence of HCV remained as independent predictors of mortality in both sexes while MELD was, prognostic factor only in the male population.

**TABLE 4:** Multivariate Cox regression model of long-term mortality prognosis.

Risk Factor	Male (C-index = 0.60)		Female (C-index = 0.64)	
	Coefficient (95% CI)	*p*-value	Coefficient (95% CI)	*p*-value
Age of recipient	1.388 (1.221–1.577)	<0.001	1.263 (1.144–1.393)	<0.001
MELD	1.210 (1.080–1.356)	<0.001	—	n.s.
Age of donor	1.321 (1.158–1.507)	<0.001	1.480 (1.350–1.624)	<0.001
Number of retransplants transplants= 1	Ref	—	Ref	—
2	2.132 (1.587–2.862)	<0.001	1.746 (1.434–2.126)	<0.001
≥3	11.834 (3.977–35.209)	<0.001	5.953 (3.768–9.405)	<0.001
HCV-negative	Ref	—	ref	—
HCV-positive	1.524 (1.291–1.789)	<0.001	1.964 (1.738–2.219)	<0.001

ref, reference category; n.s., non significant.

In Table 5, we show the results for the predictors of early mortality (1 month); number of transplants was an independent prognostic factor for mortality in both men and women. In addition recipient age, ischemia time and main disease were risk factors in the male population whereas MELD was a risk factor among females.

**TABLE 5:** Multivariate Cox regression model of early (1-month) mortality prognosis.

Risk Factor	Male (C-index = 0.68)		Female (C-index = 0.67)	
	Coefficient (95% CI)	*p*-value	Coefficient (95% CI)	*p*-value
Age of recipient	1.220 (1.074–1.387)	0.002	—	n.s.
MELD	—	n.s	1.587 (1.103–2.285)	0.010
Number of retransplant transplants				
1	ref	—	Ref	—
2	3.665 (2.834–4.731)	<0.001	2.935 (1.230–7.004)	0.010
≥3	6.048 (2.831–12.923)	<0.001	36.123 (10.986–118.780)	<0.001
Ischemia time	1.226 (1.116–1,345)	<0.001	—	n.s.
Main Disease				
Acute liver failure	5.646 (3.779–8.437)	<0.001	ref	—
Cholestasis	2.661 (1.662–4.263)	<0.001	—	n.s.
HCV positive -Cirrhosis	1.451 (1.124–1.874)	0.004	—	n.s.
HCV negative-Cirrhosis	ref	—	—	n.s.
Liver cancer	0.680 (0.507–0.911)	<0.001	—	n.s.
Other	2.235 (1.380–3.620)	<0.001	—	n.s.

ref, reference category; n.s., non-significant.

### Mortality Analysis

Mortality and overall causes of death are shown in Table 6. In our cohort, a total of 3,723 (31.5%) male patients and 1,241 (30.8%) female patients died, with important differences in the causes of mortality. The different causes of death throughout the follow-up and according to recipient sex are shown in Table 7. Surgical complications, infections, and cardiovascular diseases were the most frequent causes of mortality in the short-term while infections, recurrence of HCV-positive liver disease, and *de novo* malignancy were the most frequent causes of mortality in the long-term.

**TABLE 6:** Mortality and overall causes of death disaggregated by recipient sex.

Feature/Sex	Male (*N* = 11,914)	Female (*N* = 4,069)	*p*-value
Mortality (≤ 1 year)	1,543 (12.31%)	553 (13.71%)	**0.023**
Mortality (≤3 years)	2,214 (18.75%)	801 (19.86%)	**0.127**
Mortality (≤ 5 years)	2,692 (22.79%)	943 (23.38%)	**0.461**
Mortality (≤ 10 years)	2,908 (28.96%)	1,126 (27.91%)	**0.212**
Mortality (overall)	3,723 (31.52%)	1,241 (30.76%)	**0.379**
Cause of death (overall)			**<0.001**
Surgical complications	306 (8.37%)	104 (8.54%)	
Infection	684 (18.70%)	282 (23.15%)	
Rejection	40 (1.09%)	18 (1.48%)	
Non-malignancy recurrence HCV+	455 (12.44%)	252 (20.69%)	
Non-malignancy recurrence HCV−	62 (1.70%)	33 (2.71%)	
*De novo* liver disease	145 (3.96%)	48 (3.94%)	
Cardiovascular disease	370 (10.12%)	116 (9.52%)	
Malignancy recurrence	459 (12.55%)	88 (7.22%)	
*De novo* malignancy	561 (15.34%)	101 (8.29%)	
Renal failure	39 (1.07%)	14 (1.15%)	
Other causes	536 (14.66%)	162 (13.30%)	

Significant p values are shown in bold.

**TABLE 7:** Cause of death during follow-up by recipient sex. Data are reported as % over the entire dataset.

Cause of death/follow-up		1 year	3 years	5 years	10 years	Overall (>10 years)
Surgical complication	O	1.98%	2.27%	2.41%	2.55%	2.59%
	M	1.94%	2.23%	2.38%	2.55%	2.59%
	F	2.11%	2.41%	2.48%	2.55%	2.58%
*p*-value		0.956	0.451	0.413	0.728	0.386
Infection	O	4.03%	4.73%	5.14%	5.78%	6.07%
	M	3.80%	4.45%	4.85%	5.46%	5.79%
	F	4.69%	5.53%	5.97%	6.69%	6.99%
*p*-value		0.082	**0.002**	**<0.001**	**<0.001**	0.186
Rejection	O	0.30%	0.32%	0.35%	0.36%	0.37%
	M	0.27%	0.29%	0.32%	0.33%	0.34%
	F	0.37%	0.42%	0.45%	0.45%	0.45%
*p*-value		0.526	0.233	0.228	0.287	0.205
Non-malignancy recurrence HCV+	O	1.21%	2.52%	3.24%	4.13%	4.46%
	M	1.01%	2.20%	2.79%	3.63%	3.85%
	F	1.79%	3.45%	4.56%	5.60%	6.25%
*p*-value		**<0.001**	**<0.001**	**<0.001**	**<0.001**	**<0.001**
Non-malignancy recurrence HCV−	O	0.04%	0.14%	0.27%	0.47%	0.60%
	M	0.03%	0.12%	0.22%	0.39%	0.53%
	F	0.07%	0.20%	0.40%	0.69%	0.82%
*p*-value		0.593	0.332	0.058	**0.012**	0.671
*De novo* liver disease	O	0.44%	0.80%	0.95%	1.13%	1.22%
	M	0.43%	0.78%	0.96%	1.15%	1.23%
	F	0.47%	0.84%	0.94%	1.07%	1.19%
*p*-value		1.000	0.703	0.890	0.917	0.417
Cardiovascular disease	O	1.33%	1.79%	2.08%	2.71%	3.07%
	M	1.38%	1.84%	2.14%	2.82%	3.13%
	F	1.19%	1.64%	1.91%	2.38%	2.88%
*p*-value		0.2%	0.517%	0.707%	0.295%	1
Malignancy recurrence	O	0.64%	2.03%	2.82%	3.36%	3.45%
	M	0.74%	2.27%	3.16%	3.77%	3.89%
	F	0.37%	1.34%	1.83%	2.16%	2.18%
*p*-value		**0.007**	**<0.001**	**<0.001**	**<0.001**	**0.003**
*De novo* malignancy	O	0.35%	1.30%	2.05%	3.48%	4.18%
	M	0.42%	1.51%	2.40%	4.01%	4.75%
	F	0.15%	0.69%	1.02%	1.93%	2.50%
*p*-value		**0.010**	**<0.001**	**<0.001**	**<0.001**	**<0.001**
Renal failure	O	0.16%	0.21%	0.22%	0.29%	0.34%
	M	0.16%	0.20%	0.22%	0.30%	0.33%
	F	0.17%	0.22%	0.22%	0.27%	0.35%
*p*-value		1.000	0.934	1.000	1.000	0.246
Other causes	O	1.97%	2.57%	3.00%	3.95%	4.41%
	M	1.97%	2.56%	2.98%	4.09%	4.54%
	F	1.96%	2.60%	3.05%	3.55%	4.02%
*p*-value		0.589	0.793	0.454	0.307	0.506

O, overall population; M, male population; F, female population. Significant p values are shown in bold.

By sex, the main causes of death were infections and non-malignancy HCV-positive recurrence in females (23.2% and 20.7% of events) and infections and *de novo* malignancy in males (18.7% and 15.3% of events).

**FIGURE 3:**
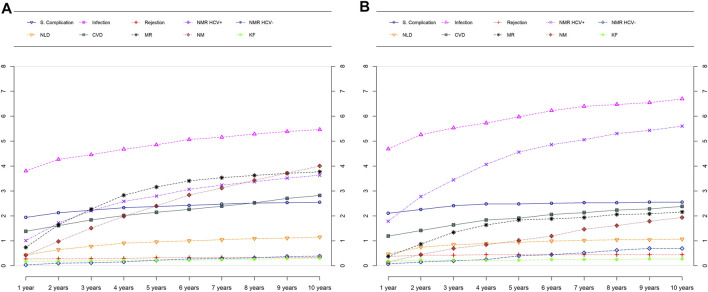
Causes of death over time by sex in males **(A)** and females **(B)**. NMR, Non-malignancy recurrence; NLD, De Novo liver disease; CVD, Cardiovascular disease; MR, Malignancy recurrence; NM, *De Novo* malignancy; KF, Kidney failure.

The cumulative relative frequency of different causes of death for male and female recipients are presented in Figure 3. Non malignancy HCV-positive recurrence (6.3% vs 3.9% of patients; *p* < 0.001) was more frequent in female than male recipients. By contrast, death because of malignancy recurrence (3.9% vs 2.2%; *p* = 0.003) and *de novo* malignancy (4.8% vs 2.5%; *p* < 0.001) were significantly more frequent in male recipients. In turn, cardiovascular disease, renal failure, recurrence of HCV-negative liver disease and surgical complications were similarly distributed as causes of death in men and women. Importantly though, 35% of women and only in 11.7% of men with mortality due to recurrence of HCV-negative disease had been transplanted for a cholestastic disease (*p* < 0.0001).

**FIGURE 4:**
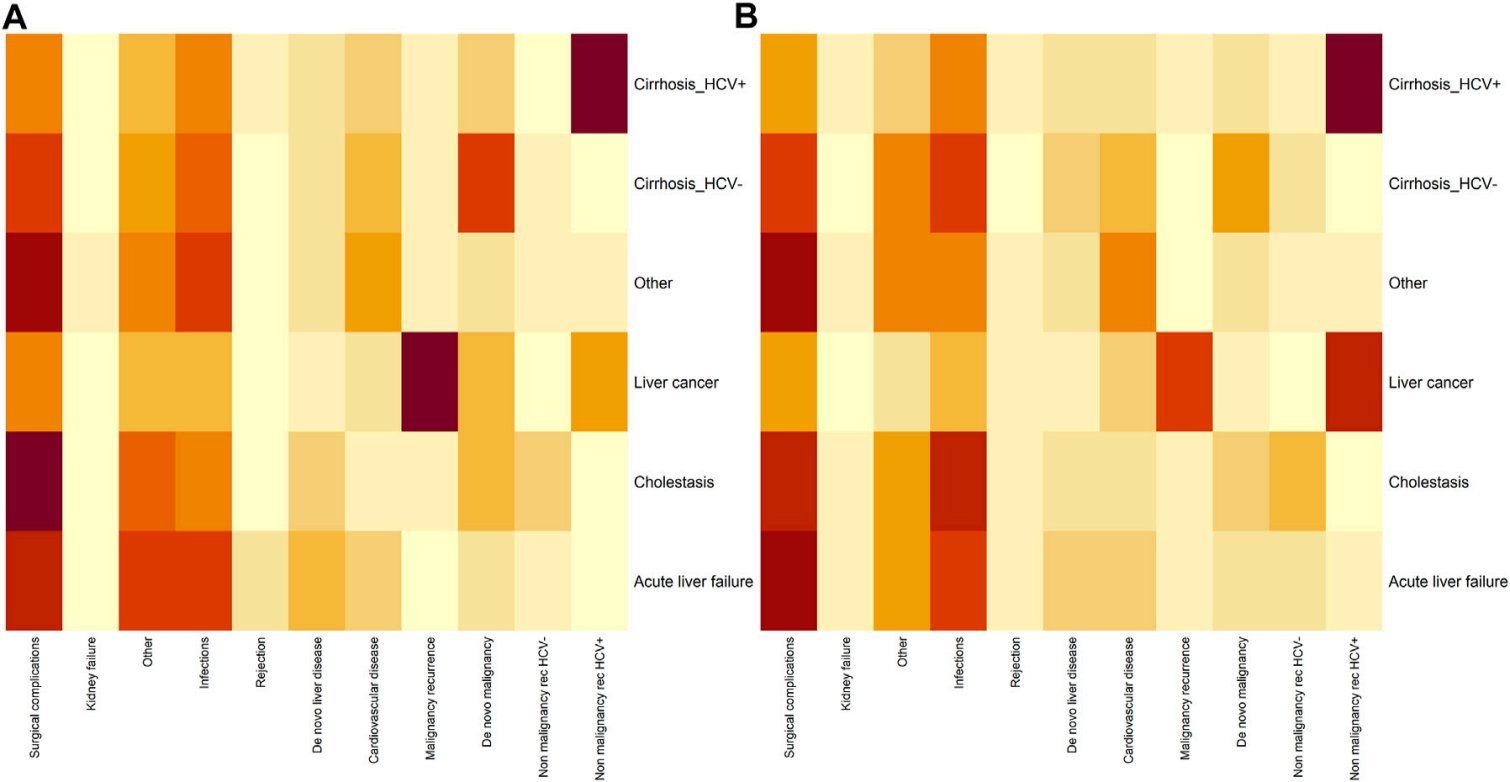
Heatmap analyzing the dependence between main disease (*Y* axis) and cause of mortality (*X* axis) in males **(A)** and females **(B)** Darker gray reflects a stronger relationship between the main disease and the cause of mortality.

We illustrate the relationship between causes of mortality and main diseases by sex in Figure 4. A heat map shows the differences by sex in the correlation between mortality and main disease; differences can be appreciated in the gradation of the color scale by sex.

## Discussion

Our study analyzed mortality data disaggregated by sex after LT in a very large sample of patients with long follow-up. We found that patient survival varies significantly according to recipient sex and the time after LT. Male patients have lower short-term mortality than females but higher long-term and overall mortality. In addition, the post-LT mortality risk related to previous liver disease is different between male and female patients, with different causes of mortality.

### Differences in Survival After Liver Transplantation

Our data show that although women have a significantly increased risk of early mortality after LT, with an overall 18% higher probability of dying in the first month after LT than males, they have better long-term survival, with males having a 6% overall higher probability of dying compared to females. A recent study based on the European Transplant Registry reported longer survival of transplanted women but did not find differences in short-term survival (9). In contrast, similar to the present study, Bruns et al. (10) reported higher mortality in women in the short-term after LT (OR 3.2), particularly among women with high MELD scores. Our results do not show a different impact of MELD according to sex on short-term mortality.

The multivariate analysis of risk factors for overall mortality found similar prognostic factors, with few exceptions. This may indicate that other factors not included in our registry, such as previous comorbidities and lifestyle, likely play an important role in mortality, mainly in the long-term.

### Mortality Risk Related to Previous Liver Disease

On the other hand, there are important differences in the etiology of liver diseases (11) that may explain, in part, some of the differences in mortality. Different liver diseases have different outcomes after LT, but the role of sex in the prognosis of these diseases has not been thoroughly evaluated. Our findings demonstrate that differences exist in this context. For example, males have 50% increased 1-year mortality when LT is performed for acute liver failure and 37% increased overall mortality when it is due to HCV-negative cirrhosis, whereas females have approximately 15% increased overall mortality when the liver disease is HCV-positive cirrhosis. This finding was expected because more severe HCV recurrence and related mortality has been described in women after LT (12–14). However, HCV-related outcomes, including LT, have changed dramatically since the emergence of new antivirals (15). Data collection in our study extended until 2017, so the effect of these drugs on survival could not be observed, but it will undoubtedly be demonstrated in the analysis of subsequent years.

Conversely, outcomes of HCV-negative cirrhosis are worse in male than in female patients. In Spain, the leading etiology in patients with HCV-negative cirrhosis is alcohol related liver disease (ALD) (16). Tobacco use, sedentary lifestyle, and unhealthy diet are often associated with alcohol consumption, and all of them are risk factors for both cardiovascular and cancer mortality. ALD patients have been shown to have excess all-cause mortality, mainly mortality related to cardiovascular disease and cancer (17), and this excess mortality is higher in males than in females. In a large Danish cohort, Salhman et al. (18) found a significant excess of different cancers in males with ALD, with an overall standardized incidence ratio of 3.01 in males and 2.33 in females (*p* < 0.001). Other findings, such as higher mortality risk in transplanted males due to acute liver failure, are more unexpected. Although acute liver failure affects females more than males and it is also associated with lower short-term survival after LT, men have a greater probability of dying when being transplanted because of this indication. Several studies have investigated the outcomes of LT in patients with acute liver failure in Western countries (19,20), but only Nephew et al. analyzed mortality according to recipient sex in the UNOS database (21). They found differences in 1-year mortality, which was no longer significant when recipient age and underlying etiology were added to the model.

Our data combined with prior studies demonstrate that mortality risk after LT related to different liver diseases varies according to sex. This is an important finding that should be considered when designing post-LT survival models.

### Causes of Mortality

Though causes of mortality have been described throughout the transplant follow-up, no sex-disaggregated analysis has been published previously. As in the non-transplanted population, there are important differences in the causes of mortality between men and women. Overall, infections are the most frequent cause of mortality in males and females, though they are significantly higher in females.

In our cohort, the main causes of mortality within the first year after transplantation were infections and surgical complications in both sexes. Although females were more frequently retransplanted, mortality due to surgical complications was similar in both. In contrast, death related to infections was significantly more common in females than in males and was evenly distributed across the different causes of liver disease, except for liver cancer. This may be explained by the clinical situation at the time of LT, crucial in explaining mortality from infections in the short-term (22). Differences in the prevalence and severity of infections between males and females vary depending on type of infection (23). Women have higher mortality in influenza A outbreaks (23), whereas male sex is a risk factor for developing severe SARS Cov-2 infection or sepsis (24,25). It seems that both immunological and hormonal factors play a role in these differences.

More differences were found in short-term mortality. Mortality because of recurrence of HCV infection was significantly higher in females, and mortality due to recurrence of hepatocarcinoma and *de novo* cancer was more frequent in males.

These differences increased with follow-up, so that in the long-term (>10 years), mortality due to infections, including HCV recurrence, was 40% higher in women than in men and mortality due to *de novo* neoplasms was almost twice as high in men as in women. Though the latter accounted for more than 15% of mortality in males, it accounted for only 8.3% in females. When we added mortality because of tumor recurrence, cancer was the leading cause of overall mortality in males, accounting for 27.9% of events and a cumulative relative frequency of 8.6% of patients, but it was the third leading cause of death in women (15.5% of events) and approximately half of the cumulative relative frequency. Hepatocellular carcinoma (HCC) is overrepresented in males, resulting in a higher number of deaths because of HCC recurrence among this population. Nevertheless, higher recurrence risk was also recently described among males. Cullaro et al. found an independent effect of sex on the risk of HCC recurrence post-LT (26). Mortality because of liver cancer recurrence increases in the first 6 years after LT and subsequently stabilizes, whereas mortality due to *de novo* cancer follows an upward trend over time.

Circulatory diseases and kidney disease are important, but not different causes of death after LT in men and women. Approximately 3% of patients globally die from circulatory disease after LT and slightly more than a third of them die in the first year after LT. A careful analysis of cardiovascular risk factors before transplantation is mandatory, as detecting patients at risk of early mortality from circulatory disease is important to avoid futile transplantation.

As expected, we found an association between some causes of mortality and certain liver diseases prior to LT. For women, the strongest association was found between acute liver failure and mortality due to surgical complications. HCV cirrhosis was associated with mortality due to non-tumor recurrence in both men and women. However, when the transplant was due to liver cancer, the strongest association was found between mortality due to tumor recurrence in men and non-tumor recurrence in women.

Mortality in LT patients is mainly related to immunosuppression. Both infections and cancer, two sides of the same coin, are related to immunosuppressive treatment. However, our data show that they are distributed differently in both sexes. Though infections result in higher mortality among females, neoplasms affect predominantly males. Knowledge of these differences is important to improve the management of patients in both the short- and long-term. In recent years, special immunosuppression protocols and surveillance programs have been proposed for the prevention or early detection of *de novo* cancer (5, 27). These results could be important to designing suitable and more cost-effective protocols according to the sex of the recipient.

Finally, although it was not the objective of our research, the imbalance found between male and female transplant recipients is remarkable. Many end-stage liver diseases affect predominantly males, and sex differences among transplant patients have been increasing over the years. From 2000 to 2016, only j 25.5% of LT patients were female. Sex differences in our registry are higher than described in other registries (9,28). These differences could reflect disparities in listing patients or in waiting-list mortality (8, 29,30). Further studies are needed to clarify this. LT is a medical process strongly influenced by sex and gender issues such that disaggregated analyses at all levels of the procedure should be mandatory to avoid disparities.

The limitations of the present study are mainly derived from its retrospective nature. Although the data entered in RETH were standardized and periodically audited, the information, as well as the consistency between sites, cannot be guaranteed. As with most studies using data from record collections, the current study may have been susceptible to practice variations and incompletely reported covariates. In addition, the definitions for causes of death may vary due to different interpretations between different teams. However, the data source is a national registry with a large number of cases that allows robust statistical analyses of a nationally representative dataset. On the other hand, due to the difficulty of national registries to rapidly adapt to changing epidemiological scenarios, we have not been able to analyze the impact of new diseases such as non-alcoholic steatohepatitis (NASH) on post-LT prognosis and causes of death. Thus, sex differences in this increasingly important disease could not be analyzed.

In summary, short- and long-term mortality and their causes are different between male and female liver transplant recipients. The risk of mortality after LT associated with different liver diseases also varies by sex. These findings are important and highlight the need for sex and gender-disaggregated analyses of clinical data.

## Spanish Liver Transplant Registry Representatives

Carolina Almohalla, Gerardo Blanco, Andrea Boscà, Federico Castillo, Ramón Charco, Valentín Cuervas-Mons, Juan Fabregat, Carmen García, Miguel Ángel Gómez, Loreto Hierro, Carmelo Loinaz, Enrique Moneva, Antonio Poyato, José Ignacio Rivas, Gonzalo Rodríguez, Fernando Rotellar, Francisco Sánchez, Julio Santoyo, Santiago Tomé, Andrés Valdivieso, Juan José Vila, Jesús Villar

## Data Availability

The data analyzed in this study is subject to the following licenses/restrictions: Are datasets belonging to Spanish Liver Transplant Society and managed and administered by the National Transplant Organization. Requests to access these datasets should be directed to www.ont.es.

## Abbreviations

ALD, alcoholic liver disease; CVD, cardiovascular disease; HCC, hepatocellular carcinoma; HCV, hepatitis C virus; HR, hazard ratio; IQR, interquartile range; KF, kidney failure; LT, liver Transplantation; MELD, model for end-stage liver disease; MR, malignancy recurrence; NLD, *De Novo* liver disease; NM, *De Novo* malignancy; NMR, non-malignancy recurrence; OR, odds ratio; RETH, Registro Español de Trasplante Hepático.
